# Nutrition and Physical Activity in Aging, Obesity, and Cancer

**DOI:** 10.1111/j.1749-6632.2012.06733.x

**Published:** 2012-10-10

**Authors:** James E Womack, Hyun-Jun Jang, Mi Ok Lee

**Affiliations:** 1 Department of Agricultural Biotechnology, Seoul National UniversitySeoul, Korea; 2 Department of Veterinary Pathobiology, Texas A & M UniversityCollege Station, Texas

**Keywords:** genomics, complex traits, animals, disease resistance, GWAS

## Abstract

The analysis of complex genetic traits, including mapping and identification of causative genes, has long been an enigma of genetic biology, whether in the animal sciences or in medical sciences. Traits of agricultural interest and traits of medical interest are often under the influence of both environmental factors and multiple genes, each with modest contributions to the total variance in the trait. Although the number of known mutations underlying complex traits is still relatively small, advances in genomics have greatly enhanced traditional pathways to their analysis and gene mining. The candidate gene approach, linkage analysis, and association studies are all significantly more powerful with recent advances in genome mapping, sequencing, and analysis of individual variation. Avenues to gene discovery are discussed with emphasis on genome wide association studies (GWAS) and the use of single nucleotide polymorphisms (SNPs) as revealed by increasingly powerful commercially available microarrays.

## Introduction

A phenotype with known or suspected genetic involvement that does not conform to classical Mendelian inheritance is generally described as a complex genetic trait. In truth, all genetic traits are complex because identical phenotypes among individuals carrying identical alleles at a given locus are rare or nonexistent, due to a number of causes including incomplete penetrance, genetic heterogeneity, and most often, a genetic background consisting of multiple genes modifying the phenotype with minor effects. Generally, however, we reserve the term *complex* for phenotypes that are influenced by multiple genes (polygenic) as well as environmental factors. Many of the traits important to animal agriculture fall into this class and are described quantitatively rather than qualitatively. Complex traits also include phenotypes that are discrete but are only expressed when the effects of multiple genes and/or environmental factors achieve a minimal threshold. These so-called threshold traits include many human diseases such as cancer and diabetes. Susceptibility to specific infectious diseases, a trait important to both animal and human health, falls in this class as well.

Genetic influence has been demonstrated for thousands of traits in domestic animals and humans. The strength of the genetic component of a trait is usually expressed as a heritability estimate for quantitative traits including many economically important traits in animals, and as familial risk factors for threshold traits, including many complex diseases in humans. Although large numbers of genomic loci underlying complex traits have been mapped, very few specific mutations have been identified and proved to be causative.

## Genome projects and genomic resources

In addition to providing a complete human genome sequence, along with cataloging of a wealth of naturally occurring genomic variation, the Human Genome Project spawned genome projects for a large number and wide variety of other organisms, including most of the common domestic animal species. Single nucleotide polymorphisms (SNPs) are the most common form of genetic variation in most species, and animal genomes appear to be replete with these, small insertions and deletions (indels), and copy number variants (CNVs). sbSNP, a database for short genetic variations, lists more than 60 million entries for humans, 9.5 million for cattle, more than 3 million for dogs and chickens, 1 million for horses, and 0.5 million for pigs as of May 16, 2012 (http://www.ncbi.nlm.nih.gov/projects/SNP/). Among the many valuable scientific resources to come from these genome projects are microarrays which permit the screening of individuals for hundreds of thousands of genomic variants in a single assay. These so-called SNP chips and CNV arrays are commercially available for humans and the common domestic species. One chip for human variants, for example, contains markers for more than 900,000 SNPs and 900,000 CNVs. A high-density cattle SNP chip contains almost 800,000 markers.

## QTLs, QTNs, and QTMs

Identification of genes responsible for monogenic traits is becoming relatively straightforward with the resources available to today's scientists. GeneCards, a database of human disease genes (http://www.genecards.org/cgi-bin/listdiseasecards.pl?type=full) lists 5,600 genes that are associated with human diseases, most of them monogenic (listing as of May 16, 2012). Online Mendelian Inheritance of Man (http://omim.org/) contains information on all known Mendelian disorders and over 12,000 genes. Even in these substantial databases, only a few genes underlying complex human disease are known.

Considerable effort in domestic animal research has gone into mapping quantitative trait loci (QTLs), the chromosomal regions (locations) that contain genomic elements contributing to variation in a trait. These studies, initially using highly polymorphic microsatellites and more recently SNPs as markers, have been highly successful. The AnimalQTLdb (http://www.animalgenome.org/cgi-bin/QTLdb/index) cites several thousand QTLs for pigs, cattle, chickens, sheep, and rainbow trout as summarized in Table 1.

**Table 1:** Summary of quantitative trait loci in animals used in agriculture

Species	No. of QTLs	No. of publications	No. of traits
Pigs	6,818	290	585
Cattle	5,920	330	407
Chickens	3,162	158	270
Sheep	684	78	189
Rainbow trout	73	8	11

From Animal QTLdb (http://www.animalgenome.org/cgi-bin/QTLdb/index), release 17, April 20, 2012.

Despite the great success in mapping QTLs, only a handful of the actual mutations responsible for trait variation have been identified. While selective animal breeding can be practiced with phase-known linked markers, identification of the genes and their specific mutations should be the scientific objective of QTL studies. Only through knowledge of genetic and cellular mechanisms accounting for variation, can phenotypes be modulated via environment, diet, vaccines, therapies, or perhaps genetic modification. While the term quantitative trait nucleotide (QTN) has been valuable in describing the nucleotide variation underlying a QTL,^1^ Andersson^2^ has introduced a more generally useful term in quantitative trait mutation (QTM), which accounts for the fact that the variation may be caused by a mutation larger than a single nucleotide.

## Candidate genes

The most intuitive approach to identification of specific genes and mutations responsible for phenotypes is the candidate gene approach. Prior knowledge of the function of a gene, coupled with some understanding of the physiological basis of the trait, has been a historical first approach to the discovery of gene/trait relationships. This logic has been exploited effectively for a number of monogenic human disease genes as well as some important phenotypes in domestic animals. As pointed out by Georges,^3^ however, the candidate gene literature is replete with nonreproducible results. Georges^3^ makes a rational plea for higher standards in candidate gene analysis, including (a) more rigid significance thresholds, (b) thorough characterization of the haplotype diversity and structure in the study population, (c) replication of positive associations in an independent cohort, and (d) resequencing individuals from functionally distinct haplotypes to find causative SNPs. As is usually the case, Michel Georges’ advice should not go unheeded.

## Linkage studies and the positional candidate approach

As discussed above and demonstrated in Table 1, a large number of QTLs have been mapped in domestic animal species, especially those of agronomic significance but only a few QTMs have been discovered. The *positional candidate approach* to gene discovery employs linkage mapping to map a trait, and then searching for most likely candidates among the genes known to lie in this region. While this has been effective in both human and animal genetics for monogenic traits, it has had limited success for QTLs. One reason is that the function of most genes is still unknown. Another is that mapping resolution is usually severely limited. A typical confidence interval in a QTL mapping study might be 20 or more centimorgans, corresponding to roughly 20 million base pairs and potentially 200 or more genes. As discussed by Georges,^3^ several factors limit mapping resolution. These are (a) marker density (which is no longer a problem in most species with the availability of SNP technology), (b) crossover density because only recombinants provide map information, (c) QTL detectance, the accuracy of inferring the genotype/phenotype relationship at loci with small contributions to the total phenotype, and (d) the molecular architecture of the QTL, that is, linked QTMs. A classic example of success with linkage analysis is the discovery by Van Laere *et al.* of the causative mutation for muscle growth and fat deposition in pigs.^4^ Identical by Descent (IBD) mapping revealed a noncoding QTN in an intron of the IGF2 gene that abates binding of a repressor and results in higher IGF2 expression in skeletal and heart muscle. This was the first noncoding QTN reported in any species. Most successful mapping studies have enhanced crossover density, often by utilizing historical recombinants and the knowledge of linkage disequilibrium (LD) and haplotype structure in the targeted species and study populations.

## Genome-wide association studies

Following the success in humans,^5^ animal geneticists can now bypass the mapping steps to a large extent in the search for QTLs and eventually the underlying QTMs. As mentioned above, high-density SNP chips covering genomes with tens to hundreds of thousands of SNPs are now available for the most common domestic animal species.^2^ Genome-wide association studies (GWAS) have been prolific in humans, where a catalog of published GWAS^6^ (http://www.genome.gov/gwastudies) includes 1,260 publications and 6,408 SNPs as of May 16, 2012. As pointed out in several reviews,^2,3,7^ the power to mine mutations underlying QTLs for complex traits is much better in domestic animals than in human populations. While domestic animals have experienced the evolution of diverse phenotypes, their young history of approximately 10,000 years permits powerful genetic dissection of phenotypic diversity.^2^ It is now well established that the LD in most domestic species is more extensive than in humans, thus permitting GWAS studies with fewer markers. Moreover, a definition of haplotype structure in different breeds or breeding populations can be exploited to reveal associations with far fewer individuals than is required in a human study. In dogs, with exceptionally high LD, power calculations have suggested that as few as 100 cases with an equal number of controls will detect loci that give a fivefold risk to a complex trait.^8^ In fact, a monogenic trait, hair ridge in Rhodesian and Thai Ridgeback dogs was mapped and the causative mutation identified from an association study with 10 cases and 10 controls.^9^

Other domestic animals have benefitted from SNP technology and GWAS as well. A number of genes for monogenic traits have been mined in horses, along with some for QTLs. GWAS in four different breeds identified a common haplotype associated with *in vitro* CD3^+^ T cell susceptibility to equine arteritis virus.^10^ Another study using GWAS with SNPs confirmed a variant in the equine myostatin gene as a predictor of racing distance in thoroughbreds.^11^ While GWAS has been used mostly for milk production traits, meat quality traits, and reproduction traits in cattle, several studies have tackled the complex traits of resistance/susceptibility to infectious diseases. These include tuberculosis susceptibility in Holstein-Fresians^12^ and susceptibility to paratuberculosis infection and Johne's disease^13–17^ in several breeds of cattle and sheep. Neibergs *et al.* have found loci on cattle chromosomes 2 and 26 linked with bovine respiratory disease and associated with persistent infection of bovine viral diarrhea virus.^18^ In pigs, several studies have targeted meat quality, fatness, and reproductive traits, and one has identified candidate genes for *E. coli* susceptibility.^19^ GWAS in chickens have focused on both meat production^20,21^ and egg production.^22,23^

## Conclusions

Complex traits remain an enigma in both human and animal genetics. While large numbers of QTLs underlying complex traits are known, the causative mutations remain elusive. However, the recent advances in genome sequencing projects, particularly the discovery of vast numbers of SNPs and their availability in affordable arrays, have opened the door to a new era in gene discovery. These tools, especially when used in GWAS, offer more than a faint hope of discovery of large numbers of genes contributing to complex traits in the very near future. The promise is especially important to studies in host resistance to infectious diseases, an important new frontier in animal and human health.
